# Understanding the environmental health implications of tourism on carbon emissions in China

**DOI:** 10.3389/fpubh.2025.1550395

**Published:** 2025-03-25

**Authors:** Jinhua Shao, Sheng Fang, Meiling Zhao, Wanxin Qian, Cai Wang

**Affiliations:** ^1^School of Humanities and Social Sciences, Anhui University of Science and Technology, Huainan, China; ^2^College of Tourism, Hainan Tropical Ocean University, Sanya, China

**Keywords:** tourism industry, carbon emission, machine learning, decoupling index, environmental health

## Abstract

Tourism development is important for the formulation of the national carbon reduction policy. China has put forward the goals of carbon peaking and carbon neutrality. Studying the impact of China’s tourism industry on carbon emissions is of great significance in scientifically formulating emission reduction policies and helping China to realize its carbon reduction goals. In this study, we simulate the complex relationship between the tourism industry and carbon emissions in China using machine learning models. This study is the first to employ interpretable machine learning to analyze the impact of the tourism industry on carbon emissions in China. Our findings demonstrate that sparrow search algorithm and random forest (SSA-RF) hybrid model can model the relationship between carbon emissions and tourism factors with low error. The expansion of the tourism industry positively contributes to the increase in carbon emissions. Our study highlights the need to consider tourism factors when formulating national carbon reduction policy.

## 1 Introduction

Sea level rise and extreme weather events caused by global warming are a serious threat to the sustainable development of society (1, 2), and reducing carbon emissions is essential for controlling global warming. China’s role in mitigating global change and reducing emissions has become even more important after the United States withdrew from the Paris Agreement (3, 4). China is the world’s largest carbon emitter (5), and the Chinese Government is actively taking measures to reduce emissions and has formulated targets for such reductions (6, 7). China proposes to achieve carbon peaking by 2030 and carbon neutrality by 2050. These targets are not only significant for China, but also play an important role in slowing down global warming.

According to a report released by the International Energy Agency, carbon emissions from the tertiary industry grew at a rate of up to 50.43% from 2011 to 2018, which is an important contributor to the growth of carbon emissions, and the growth rate of carbon emissions from the tertiary industry is much larger than that of the primary and secondary industries. Tourism is an important part of the tertiary industry (8), and the 2021 United Nations Climate Change Conference formally passed the Glasgow Declaration, which emphasized practical and strong measures to help achieve the global commitment to zero carbon emissions by 2050. Most of the analysis of the factors affecting carbon emissions in the tourism industry uses decomposition analysis (9–11), which includes structural decomposition analysis (SDA) and index decomposition analysis (IDA). Using decomposition method, total domestic tourists’ economic yield proved to be the largest driver of carbon emission growth in China’s tourism industry, and total international tourists’ economic yield also contributed to the growth of carbon emissions in China’s tourism industry. Energy consumption intensity and energy consumption structure, on the other hand, are important factors in slowing down the carbon emissions of the tourism industry (9). There are other factors that have also contributed the growth of carbon emissions in China’s tourism industry, such as population and economic development (12). The factors influencing carbon emissions from the tourism industry are relatively well researched (13).

In recent years, more and more scholars have begun to focus on how tourism affects a region’s total carbon emissions, but there are no conclusive findings on how tourism affects a region’s total carbon emissions. Existing studies show that tourism reduces carbon emissions in Europe, but in Asia it contributes to the growth of regional carbon emissions. According to Lee and Brahmasrene (14), the tourism industry in Europe exerts a significant negative impact on carbon emissions. At the global scale, most studies show that tourism contributes to global carbon emissions (15). Air transport and visitor spending have hindered Australia’s progress toward achieving net-zero emissions (16). The relationship between tourism and regional carbon emissions is complex (17, 18). Tourism development has an obvious role to play in boosting a region’s economy, but tourism development may either promote or inhibit regional carbon emissions. Therefore, it is necessary to analyze the impact of tourism development on regional carbon emissions before deciding whether or not to mitigate carbon emissions by developing tourism. Currently, analyzing the impact of tourism on the region’s carbon emissions also mainly uses decomposition analysis. Visas et al. (19) analyzed the impact of tourism on energy consumption in the BRICS using decomposition method, the results reveal that the tourism promoted the energy consumption.

Over the past several years, the influence of artificial intelligence (AI) has expanded rapidly (20), and AI has been widely used in establishing carbon emission prediction models (21), with numerous studies demonstrating that AI models outperform traditional approaches in carbon emission forecasting (22). This is due to the fact that AI models are better able to capture the complex relationship between carbon emissions and influencing factors than traditional regression models. Despite the high prediction accuracy of the AI models, the models are poorly interpretable and have difficulty explaining the effects of variables on carbon emissions. Many cutting-edge studies in the fields of chemistry (23), biology, and materials science (24) use machine learning models to explain the results of their studies, which are referred to as interpretable machine learning models in research. Interpretable machine learning models are not only a data fitting tool but also an analytical tool in research. We believe that interpretable machine learning techniques have great potential to reveal the complex relationship between tourism and carbon emissions. Although many previous papers have examined the relationship between carbon emissions and tourism, there are few studies using interpretable machine learning, and we are interested in filling a gap. Overall, the main goal of this paper is to analyze the impact of tourism factors on carbon emissions using interpretable ML, emphasizing that the development of tourism should also focus on the impact on global warming.

## 2 Methodology

### 2.1 Data sources

China’s carbon emissions data for 2000–2019 is from BP’s World Energy Statistics. The data on international tourism revenue (ITR), number of international tourist arrivals (ITA), foreign exchange earnings from international tourism transportation (ITT), total domestic tourism expenditure (DTE), and number of domestic tourists (DT) are sourced from the National Bureau of Statistics of China. We processed some of the missing data using autoregressive integrated moving average (ARIMA) method. ARIMA is a widely used statistical model for time series analysis and forecasting, and it is also widely used in data interpolation (25). Compared to other interpolation methods such as long short-term memory (LSTM) and conditional generative network (CNN), the ARIMA model is highly interpretable, whereas the internal mechanisms of LSTM and CNN are complex, and the LSTM and CNN models require a large amount of data to achieve good prediction accuracy. For this study, the data size is small, so we chose ARIMA for missing data.

### 2.2 Random forest

Random forest (RF) is a commonly used machine learning algorithm for regression prediction (26), which achieves high accuracy by constructing multiple decision trees and aggregating their prediction results. Compared to conventional deep learning algorithms, RF does not require large amounts of data and trains quickly. It has fewer hyperparameters, which are easier to adjust, whereas deep learning requires extensive time and data to train hyperparameters. Given the small scale of data in this study, using deep learning algorithms could lead to overfitting. This study uses the RF method to model the relationship between carbon emissions and the tourism industry in China. The training data for each base learner in RF is obtained through the bootstrap method, which involves randomly selecting a subset of the overall features, with the rest of the data constituting the out-of-bag samples (OOB) sample. The randomness of the algorithm is demonstrated in two ways: firstly, the training data for each tree is selected by random sampling; secondly, the tree features selected are also chosen randomly. This approach not only prevents model overfitting, but also enhances the differences between individual decision trees by adding randomness to the training process. With these steps, the constructed forest can improve the prediction accuracy by averaging the predictions of the trees as shown in the Equation 1:


(1) $$\bar{f}=\frac{1}{n}\overset{n}{\underset{i=1}{\sum }}T\left(x,O_{i}\right)$$

Where $\bar{f}$ is the average, $T\left(x,O_{i}\right)$ is the output of each tree and *n* represent the trees.

### 2.3 Optimization algorithm

The particle swarm algorithm (PSO), an optimization algorithm developed by simulating the foraging behavior of birds, is a simple and easy-to-implement optimization algorithm that is widely used in training machine learning models for hyperparameter optimization. The algorithm proposes the concept of particles to simulate birds in a flock, where particles learn and exchange information among themselves to achieve a globally optimal search. Each particle in the particle swarm algorithm has its own position and velocity, and the velocity of each example is updated according to Equations 2–4:


(2) $${v_{j}}^{\left(i+1\right)}=w{v_{j}}^{\left(i\right)}+c_{1}r_{1}\left(\mathrm{pbes}t_{j}-{x_{j}}^{\left(i\right)}\right)+c_{2}r_{2}\left(\mathrm{gbes}t_{j}-{x_{j}}^{\left(i\right)}\right)$$

(3) $$v_{\text{min}}\leq {v_{j}}^{\left(i+1\right)}\leq v_{\text{max}}$$

(4) $${x_{j}}^{\left(i+1\right)}={x_{j}}^{\left(i\right)}+{v_{j}}^{\left(i+1\right)}\;j=1,2,\ldots ,n$$

where ${v_{j}}^{\left(i\right)}$ is the velocity of particle *j* at the *i*-th iteration, and is w the inertia weight. $c_{1}$ and $c_{2}$ are the learning factors, which control how close the particle is to its own optimal position $\mathrm{pbes}t_{j}$ and the global optimal position $\mathrm{gbes}t_{j}$ . $r_{1}$ and $r_{2}$ is a random number between 0 and 1 to increase the randomness of the algorithm. ${x_{j}}^{\left(i\right)}$ is the position of the particle at the *i*-th iteration. $v_{\text{min}}$ and $v_{\text{max}}$ are the minimum and maximum velocities of the particle. Equation 4 is the formula for particle position update.

The whale optimization algorithm (WOA) is a swarm optimization algorithm proposed by simulating the feeding behavior of humpback whales. The whale optimization algorithm consists of three steps surrounding the prey, bubble net attack method and prey search.

The behavior of surrounding prey is simulated by Equations 5–8:


(5) $$\vec{D}=|\vec{C}\cdot {\vec{X}}^{*}\left(t\right)-\vec{X}\left(t\right)|$$

(6) $$\vec{X}\left(t+1\right)={\vec{X}}^{*}\left(t\right)-\vec{A}\cdot \vec{D}$$

(7) $$\vec{A}=2\vec{a}\cdot \vec{r}-\vec{a}$$

(8) $$\vec{C}=2\cdot \vec{r}$$

Where ${\vec{X}}^{*}\left(t\right)$ is the optimal solution (prey position), and $\vec{X}\left(t\right)$ is the location of the whale. $\vec{A}$ and $\vec{C}$ are coefficient vectors that change dynamically as the algorithm iterates. $\vec{a}$ is a factor that decreases linearly from 2 to 0, and $\vec{r}$ is a random forest between 0 and 1. When $\left|\vec{A}\right|$ is less than 1, the whale moves closer to the prey, and when $\left|\vec{A}\right|$ is greater than 1, the whale moves away from the prey, thus performing a global search.

The bubble net attack is the algorithm and core to simulate the bubble net hunting behavior of whales (Equation 9).


(9) $$\vec{X}\left(t+1\right)={\vec{D}}^{'}\cdot e^{bl}\cdot \cos\left(2\pi l\right)+{\vec{X}}^{*}\left(t\right)$$

where ${\vec{D}}^{'}$ denotes the current distance between the whale and the prey, b is a constant, and l is a random number between −1 and 1.

To avoid a local optimal solution, the whale population performs a global search by randomly selecting another whale and moving away from it (Equation 10):


(10) $$\vec{X}\left(t+1\right)={\vec{X}}_{\mathrm{rand}}-\vec{A}\cdot \vec{D}$$

where $\vec{X}\left(t+1\right)$ is the location of a randomly selected whale.

Sparrow search algorithm (SSA) is an intelligent optimization algorithm proposed based on the foraging and anti-predator behavior of sparrows. In the foraging process of sparrows, they are divided into finders and joiners. The discoverers are responsible for providing foraging areas and directions for the entire sparrow flock, while the joiners rely on the guidance of the discoverers for food. In addition, when a sparrow flock becomes aware of danger, it triggers anti-predatory behavior. Discoverers have better adaptations and therefore finders can gain a larger search range than joiners. The finder’s location update method can be described as follows (Equation 11):


(11) $$X_{i,j}^{t+1}=\{\begin{array}{c} X_{i,j}^{t}\exp\left(-\frac{i}{s\cdot t_{\text{max}}}\right)\;Y_{2}<\mathrm{AN}\\ X_{i,j}^{t}+ZD\;Y_{2}\geq \mathrm{AN} \end{array}$$

where $X_{i,j}^{t+1}$ is the position of the sparrow i in dimension j and $t_{\text{max}}$ is the maximum number of iterations. s is a random number between 0 and 1. $Y_{2}$ denotes the warning value, AN denotes the safety value, the value range of $Y_{2}$ and AN is 0–1 and 0.5–1, respectively. Z is a random number with normal distribution. D is the 1×d matrix, d is the dimension of the optimization problem, and all elements of the matrix are 1. When $Y_{2}<\mathrm{AN}$ , it indicates that there are no predators in the range. The finder can perform a wide search operation. When $Y_{2}<\mathrm{AN}$ , it indicates that a predator has been found. At this time, all sparrows need to quickly fly to other safe places. The method of updating joiner locations is described as follows (Equation 12):


(12) $$X_{i,j}^{t+1}=\{\begin{array}{c} Z\cdot \exp\left(\frac{X_{w}-X_{i,j}^{t}}{i^{2}}\right)\;i>\frac{n}{2}\\ X_{p}^{t+1}+|X_{i,j}^{t}-X_{p}^{t+1}|A^{+}\cdot D\;\mathrm{else} \end{array}$$

where $X_{p}$ is the current best position of the discoverer, and $X_{w}$ is the worst position of the current discoverer. A is a 1×d matrix where each element of the matrix is randomly assigned a value of 1 or − 1 and $A^{+}=A^{T}{\left(AA^{T}\right)}^{-1}$ , *n* the number of sparrows in the flock. When i>n/2 , it means that the joiner i has a low fitness value and is very hungry and needs to fly elsewhere to forage for food. When aware of the danger, sparrow populations perform an anti-predatory behavior, the mathematical expression for which is (Equation 13):


(13) $$X_{i,j}^{t+1}=\{\begin{array}{c} X_{b}^{t}+\beta |X_{i,j}^{t}-X_{b}^{t}|\;f_{i}>f_{g}\\ X_{i,j}^{t}+G\left[\frac{\left|X_{i,j}^{t}-X_{w}^{t}\right|}{\left(f_{i}-f_{w}\right)+\varepsilon }\right]\;f_{i}=f_{g}\; \end{array}$$

Where $X_{b}$ is the global optimal position, β is the parameter controlling the step size and obeys a normally distributed random number with mean 0 and variance 1. G is a random number between −1 and 1, $f_{i}$ is the fitness value of the sparrow i , and $f_{g}$ and $f_{w}$ are the global optimum and global worst value. $f_{i}$ is the smallest constant that avoids a denominator of 0 in equation.

### 2.4 SHapley Additive exPlanation

The aim of interpretable machine learning is to learn how the model make predictions, and find the relationship between the input and output, more importantly, to answer the question of which input feature is important for driving prediction results. Model interpretable method can be divided into two categories, local interpretable method and global interpretable method. Global interpretation can explain the importance distribution of features across the entire dataset, while local interpretation allows for a detailed analysis of the contribution of each feature to the prediction of a particular observation. SHapley Additive exPlanation (SHAP) enables not only local interpretation but also global interpretation, and SHAP makes use of the Shapley value concept in game theory, and by accurately calculating the contribution of each feature to the model output, it directly demonstrates the weight and influence of each feature in the model prediction (27). For feature i in the feature set S , the Shapley value is calculated as follows (Equation 14):


(14) $$\Phi_{i}=\underset{S\subseteq N\backslash \left\{i\right\}}{\sum }\frac{|S|!\left(|N|-|S|-1\right)!}{|N|!}\left(v\left(S\cup \left\{i\right\}\right)-v\left(S\right)\right)$$

where N denotes the set of all features, and S is any subset of features that does not contain feature i . |S| is the number of features in the set S . $v\left(S\right)$ is the contribution of the feature set S to the predicted output of the model. $v\left(S\cup \left\{i\right\}\right)$ is the contribution of the feature set $S\cup \left\{i\right\}$ containing feature i to the predicted output of the model.

### 2.5 Hybrid model

**Figure 1:**
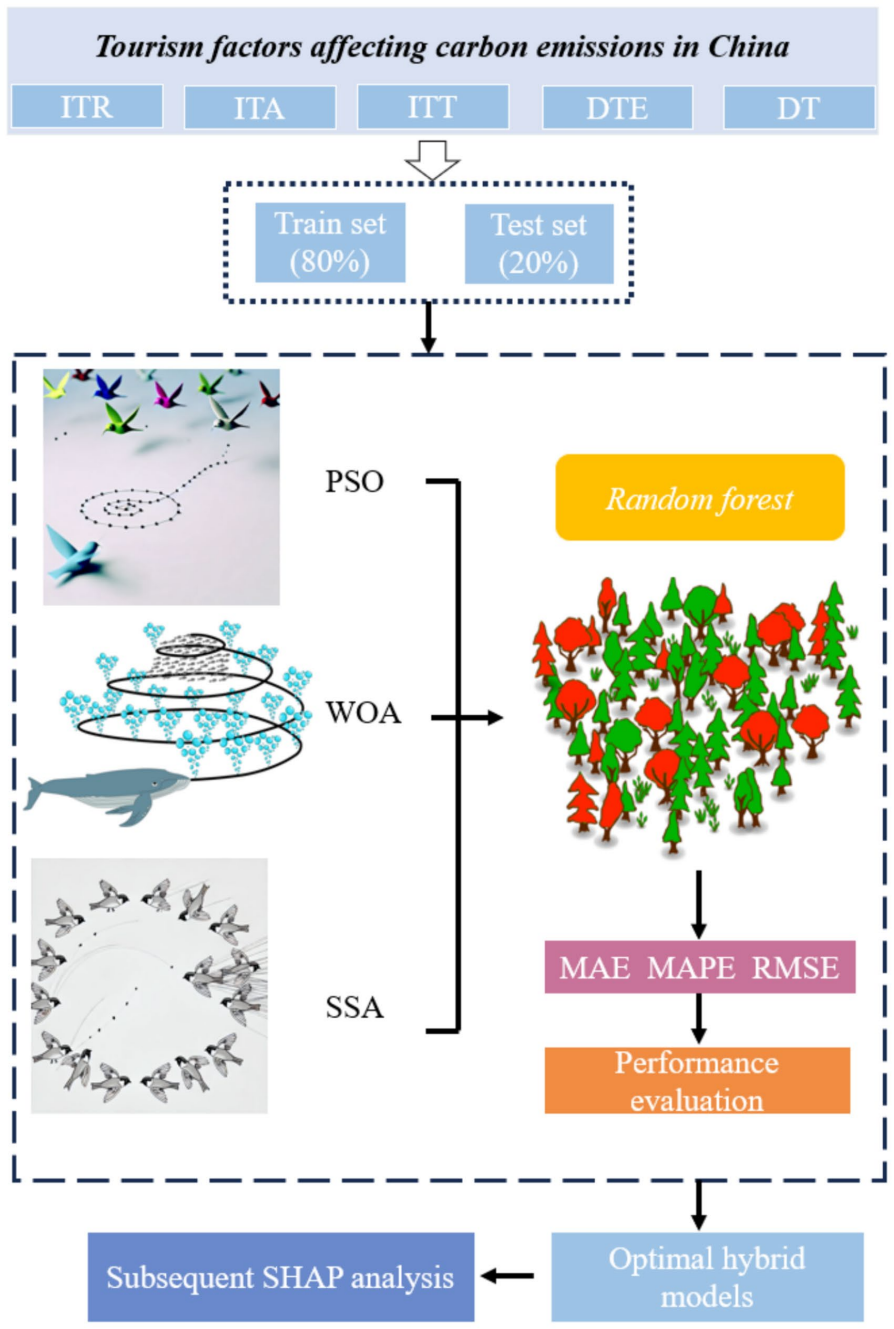
The flowchart of hybrid models.

SHAP is model-agnostic, so it can be applied to a wide range of machine learning models. The calculation of SHAP values is not directly related to the accuracy of the model, but the performance of the model may indirectly affect the stability of SHAP values. If the accuracy of model is high, it means that the model is performing well in capturing the relationship between features and outputs. As a result, the SHAP value is more reflective of the true impact of the features on the model’s predictions. In other words, in high accuracy models, SHAP values are more interpretive and results are more credible. Therefore, in this paper, the RF model is first optimized using the swarm optimization algorithm (PSO, WOA and SSA) to improve the prediction accuracy of the RF model and to ensure the stability of the subsequent SHAP analysis. We optimized the hyperparameters (n_estimators and max_features) of the RF model using PSO and SSA, and three hybrid model (PSO-RF, WOA-RF and SSA-RF) are established. The flowchart of three hybrid models is shown in Figure 1.

### 2.6 Tapio decoupling index

The carbon emission decoupling index is an index that measures the relationship between economic growth and carbon emissions (28). It aims to evaluate the relationship between economic growth and greenhouse gas emissions in a country or region, i.e., whether economic growth is accompanied by a corresponding reduction in carbon emissions (29). Currently, the Tapio model is mostly used in the analysis of decoupling relationship. Compared with the previous OECD model (30), Tapio can more comprehensively reflect the multiple and complex relationships between environmental pressure and economic development, which can better help policy makers to develop feasible strategies to achieve a balance between economic growth and environmental protection. According to the definition given Tapio, the tourism carbon emission decoupling index can be calculated as follows (Equation 15):


(15) $$T=\frac{\%\Delta CE}{\%\Delta E}=\frac{\left(CE_{t}-CE_{t-1}\right)/CE_{t-1}}{\left(E_{t}-E_{t-1}\right)/E_{t-1}}$$

Where CE is the carbon emission of China, and E is the revenue from tourism industry. ΔCE and ΔE are the change rates of carbon emissions and tourism revenues in China. The relationship between carbon emissions and tourism revenue can be divided into three major types of states: coupling, decoupling and negative decoupling, as shown in Table 1.

**Table 1:** Decoupling index classification criteria.

State		%∆*CE*	%∆*E*	*T*
Decoupling	Strong decoupling	<0	>0	T<0
	Weak decoupling	>0	>0	0≤T<0.8
	Recessive decoupling	<0	<0	T>1.2
Coupling	Expansive coupling	>0	>0	0.8≤T≤1.2
	Recessive coupling	<0	<0	0.8≤T≤1.2
Negative decoupling	Expansive negative decoupling	>0	>0	T>1.2
	Weak negative decoupling	<0	<0	0≤T<0.8
	Strong negative decoupling	>0	<0	T<0

## 3 Results and discussion

### 3.1 Optimal hybrid model selection

**Figure 2:**
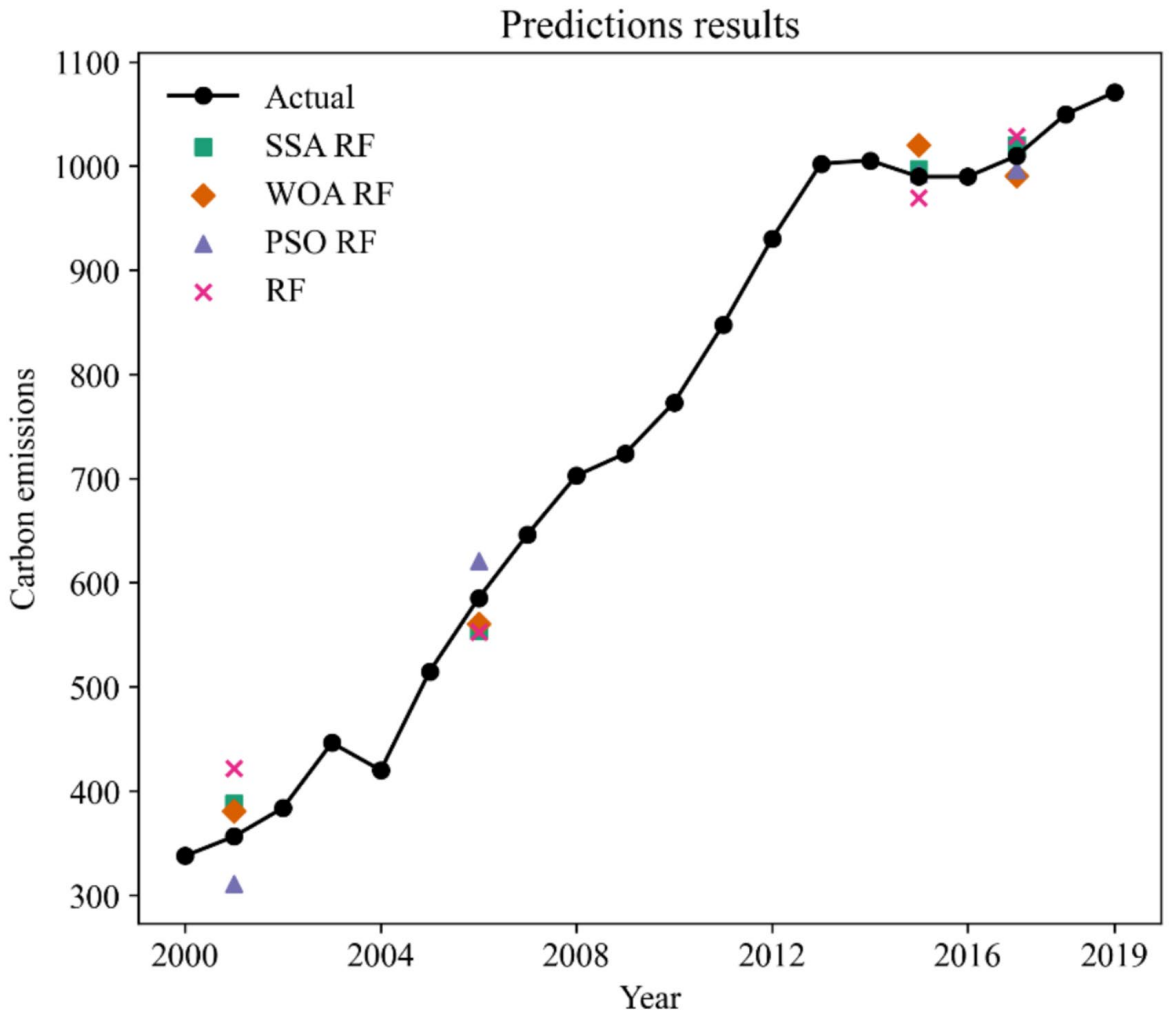
Prediction results of models.

Based on existing research and data availability (31–34), we use international tourism revenue (ITR), number of international tourist arrivals (ITA), foreign exchange earnings from international tourism transportation (ITT), total domestic tourism expenditure (DTE), and number of domestic tourists as the input to the RF model, and China’s carbon emissions are the output of the model. N_estimators and max_features are two important parameters of the RF model. Increasing the value of n_estimators usually improves the stability and performance of the model, as more trees means that the model can fit the data better. More trees also increase training time and memory consumption. Max_features values usually increase the diversity of the model, thus reducing the risk of overfitting. Small max_features value may result in a decrease in the performance of each tree, affecting the overall model performance. Conventional grid search is time-consuming, so in this study, three swarm optimization algorithms were used to optimize the parameters of RF to obtain highly accurate models. When training the model, we use 80% of the dataset as the train set and 20% of the dataset as the test set. The division of the train and test sets is random. Figure 2 shows the prediction results of the three hybrid models and the unoptimized RF model on the test set.

As shown in Figure 2, the prediction results of the SSA-RF and WOA-RF models have the smallest deviation from the actual value of carbon emissions, which indicates that the two models are better able to simulate the relationship between carbon emissions and tourism indicators. The PSO-RF has large errors in predicting carbon emission values for 2001 and 2006, but the deviation between the model predictions and the actual values is small when predicting carbon emissions for 2015 and 2017. Whereas RF is the model with the largest deviation in prediction results among the four models, the model does not work well in each year of the test set.

In order to quantitatively compare the prediction accuracy of different machine learning models, we choose Mean Absolute Error (MAE), Mean Absolute Percentage Error (MAPE), and Root Mean Square Error (RMSE) as the evaluation metrics of model performance. Evaluation metrics are calculated based on the prediction results of different machine learning prediction models, and the evaluation metrics are compared to measure the strengths and weaknesses of the prediction models. The formula for calculating the evaluation metrics are as follows:


(16) $$MAE=\frac{1}{N}\overset{N}{\underset{i=1}{\sum }}|E_{p}-E_{a}|$$

(17) $$\mathrm{MAPE}=\frac{1}{N}\overset{N}{\underset{i=1}{\sum }}\frac{\left|E_{p}-E_{a}\right|}{\left|E_{a}\right|}\times 100\%$$

(18) $$\mathrm{RMSE}=\sqrt{\frac{1}{N}\overset{N}{\underset{i=1}{\sum }}{\left(E_{p}-E_{a}\right)}^{2}}$$

In Equations 16–18, $E_{p}$ and $E_{a}$ are the predicted and actual values of machine learning models. The calculation results of evaluation metrics are shown in Table 2.

**Table 2:** Evaluation metrics of models.

	SSA-RF	WOA-RF	PSO-RF	RF
MAE	20.21	24.69	24.29	34.19
MAPE	4	4.01	5.12	6.94
RMSE	23.12	25.02	29.70	38.96

MAE represents the mean absolute difference between the predicted value and the actual value. According to the MAE, the prediction accuracy is ranked from highest to lowest as SSA-RF > PSO- WOA-RF > PSO-RF > RF. The prediction accuracies of the hybrid models are all greatly improved compared to the single RF. The prediction accuracies of models SSA-RF, WOA-RF, and PSO-RF are improved by 1, 2, and 3%. An important feature of MAE is that it is insensitive to outliers because it only focuses on the mean of the absolute error, so we need other metrics to evaluate the model comprehensively. According to the MAPE, the prediction accuracy ranked from highest to lowest is SSA-RF > WOA-RF > PSO-RF > RF. SSA-RF has the smallest MAPE value indicating that it has the best prediction performance. SSA-RF is the optimal model can also be found based on the value of RMSE. In Figure 2. We can see that both SSA-RF and WOA-RF possess good prediction results, but based on the evaluation metrics we are able to determine that SSA-RF is the best prediction model.

### 3.2 The influence of tourism factors on China’s carbon emissions

**Figure 3:**
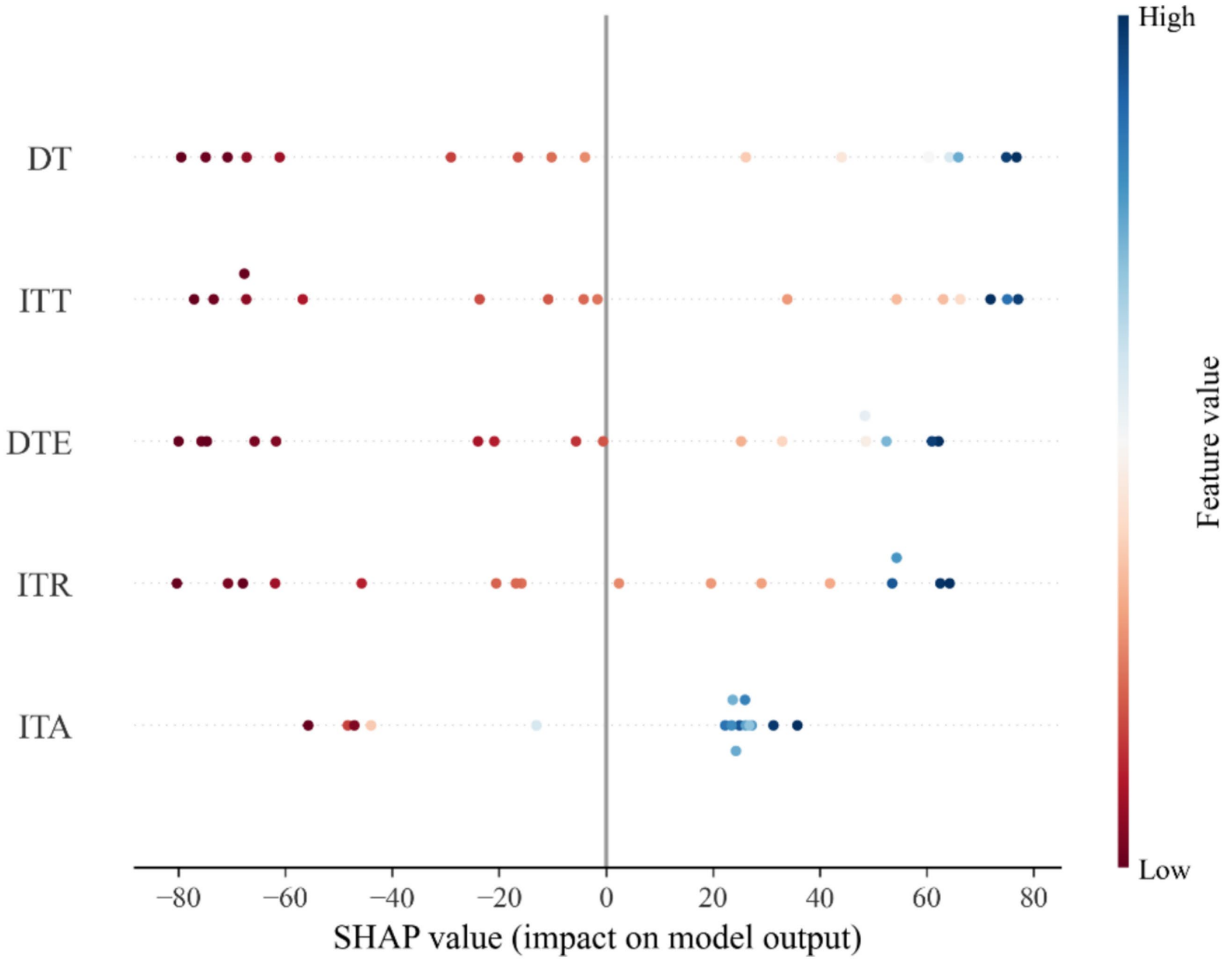
SHAP value of tourism factors.

To analyze the influence of tourism industry on carbon emissions in China, scatter plots of SHAP values are plotted as shown in Figure 3.

This figure of SHAP values shows the impact of different features on the carbon emissions. In the figure of SHAP values, the horizontal axis indicates the SHAP value (effect on carbon emission) and the vertical axis indicates the different features (DT, ITT, DTE, ITR, ITA). The color from red to blue indicate the feature values from high to low. Negative values of SHAP indicate that the feature has a decreasing impact on the carbon emissions, while positive values indicate an increasing impact. The SHAP values for DT, ITT, DTE, and ITR are uniformly distributed across both the negative and positive sides of the scale, indicating that these features can either positively or negatively influence model predictions. The uniform variation of SHAP values with changes in DT, ITT, DTE, and ITR suggests a linear effect of these features on carbon emissions in China. This uniformity implies that as the values of these features increase or decrease, their impact on the model’s prediction of carbon emissions consistently follows a linear trend, either enhancing or reducing the predicted emissions. On the other hand, the SHAP values for international tourist arrivals (ITA) are predominantly found in the positive region. This concentration in the positive spectrum indicates that ITA significantly contributes to increasing the predicted value of the model’s output, suggesting a positive relationship between ITA and carbon emissions in China.

**Figure 4:**
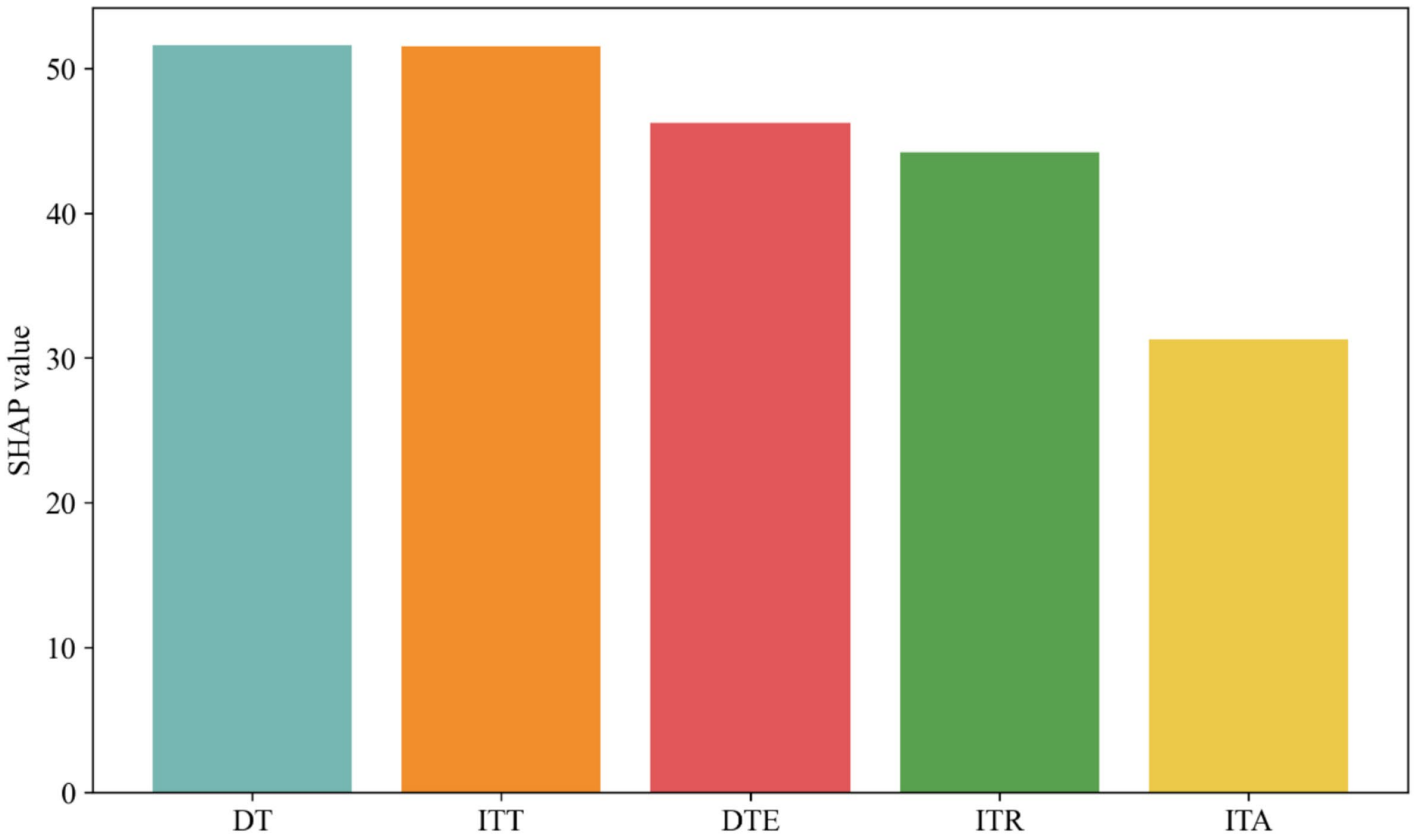
SHAP value of DT, ITT, DTE, ITR, ITA.

Figure 4 shows the average SHAP values for DT, ITT, DTE, ITR and ITA. The largest values for DT and ITT indicate that DT and ITT drive the growth of carbon emissions in China, and the smallest SHAP value for ITA indicates that ITA has the least impact on the growth of carbon emissions in China. The smallest impact of ITA on China’s carbon emissions can also be observed in Figure 3, where the SHAP value of ITA is closest to the 0-axis.

### 3.3 Decoupling analysis between tourism industry and carbon emissions

**Figure 5:**
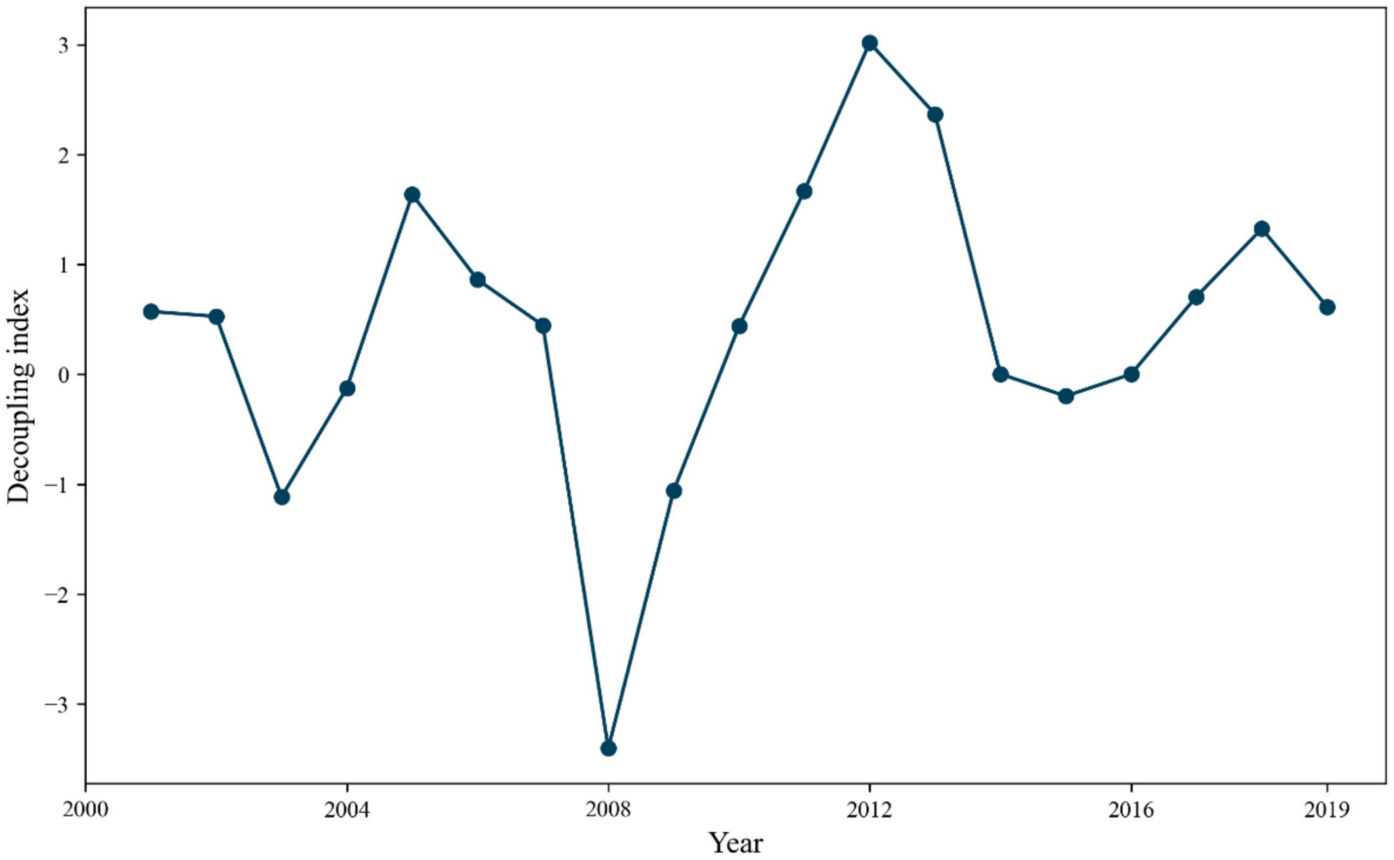
Decoupling index from 2000 to 2019.

According to the Equation 14, the decoupling index of carbon emissions and tourism in China from 2000 to 2019 is calculated, and the results are shown in Figure 5. Weak decoupling in 2001 and 2002 indicates that tourism revenue is growing faster than carbon emissions, but carbon emissions are still increasing. The shift from strong negative decoupling to strong decoupling between 2003 and 2004 suggests that economic policies or environmental protection measures during this period promoted economic growth while reducing carbon emissions. Both decoupling and coupling states existed from 2005 to 2009, and in 2009, the decoupling state was a strong negative decoupling, illustrating the fact that tourism revenues were decreasing at the same time that carbon emissions were increasing, and that the policies of this period seriously threatened the sustainability of society.

From 2010 to 2019, the decoupling state shows a steady weak decoupling, and the growth rate of tourism revenue is greater than the increase rate of carbon emissions in this period, but policy guidance should also be strengthened to focus on environmental protection while boosting tourism revenue.

## 4 Conclusions and policy recommendations

### 4.1 Conclusion

This paper firstly compares the results of different machine learning models in predicting China’s carbon emissions, and then analyzes the influence of international tourism revenue, number of international tourist arrivals, foreign exchange earnings from international tourism transportation, total domestic tourism expenditure, and number of domestic tourists on China’s carbon emissions using SHAP values. Finally, the decoupling index is used to analyze the decoupling relationship between tourism revenue and carbon emissions in China. The main conclusions are as follows:

SSA-RF is able to capture the complex relationship between tourism factors and China’s carbon emissions, and the predictions of SSA-RF model have the lowest MAE, MAPE and RMSE. Domestic tourists and international tourism transportation have the largest SHAP values, and these two factors have the greatest impact on changes in China’s carbon emissions. According to the SHAP value analysis, the growth of China’s tourism industry shows a linear relationship with the increase in China’s carbon emissions, i.e., the expansion of the tourism industry is directly proportional to the increase in carbon emissions. Over the past decade, China’s tourism revenues have continued to grow, while at the same time the trend of increasing carbon emissions has slowed down, showing a decoupling between tourism revenues and carbon emissions.

### 4.2 Policy implications

Considering the role of tourism in contributing to the growth of China’s carbon emissions, China should strongly promote a low-carbon transition in the tourism industry. Firstly, the accounting of carbon emissions from tourism should be strengthened, and the tourism industry should specify clear goals and paths for low-carbon development. Actively develop low-carbon and environmentally friendly tourism products and strengthen low-carbon and environmental protection education during tourists’ travels.

Transportation plays an extremely important role in the low-carbon transition of tourism, as confirmed in our study. Governments should encourage the use of public transportation by tourists, for example, by installing bicycle rental stations and walking paths in tourist destinations, and by improving orientation information to help tourists make better use of these services. In regional tourism, resources can be shared through multi-regional cooperation to jointly promote low-carbon transportation strategies, such as the construction and operation of cross-regional public transportation systems.

The transportation process for foreigners traveling into China also has a significant impact on China’s carbon emissions, and carbon emissions from air transits can be reduced by cooperating on direct flights to popular tourist destinations. Increase multilingual signage on public transportation and actively guide foreign tourists to use public transportation.

## Data Availability

The original contributions presented in the study are included in the article/supplementary material, further inquiries can be directed to the corresponding authors.
